# Sœmmerring's error: the root of the story. The C8 nerve is a misconception. A historical review and anatomical perspectives

**DOI:** 10.3389/fnana.2025.1568824

**Published:** 2025-04-10

**Authors:** Patrick Chaynes, Luana Carfagna, Marina Poinsignon, Amaury De Barros

**Affiliations:** ^1^Department of Anatomy and Surgical Simulation, Toulouse Medical School, Toulouse Federal University, Toulouse, France; ^2^Service of Neurosurgery, Neurosciences Department, Toulouse University Hospital, Toulouse, France; ^3^Department of Pediatric Surgery, HÃ'pital des Enfants, Toulouse University Hospital, Toulouse, France

**Keywords:** spinal nerve, history, somites, metameres, history of anatomy

## Introduction

Which doctor did not learn the arrangement of the spinal nerves? (*I did, and I'm sure you did too*). Which medical students did not ask: “How can there be eight cervical nerves when there are only seven cervical vertebrae”? (*I did, and maybe you did too*). The answer is always the same: “My dear young student, this discrepancy is easily explained. The first cervical nerve runs superior to the first cervical vertebra, the atlas, and the last runs between the seventh cervical and the first thoracic vertebrae, leaving six cervical nerves in between. So, there are eight in number.”

In the same way, the 42nd British edition of *Gray's Anatomy* (Standring, 2020), along with every single anatomical textbook worldwide, reports 31 pairs of spinal nerves, arranged as follows: eight cervical, 12 thoracic, five lumbar, five sacral and one coccygeal.

This fundamental assumption is so deeply ingrained that it would be considered sacrilegious to have doubts about it, as it appears to be an incontestable certainty. Anyone who dares to contradict it risks being seen as either presumptuous or foolhardy, but that ordering, established in the late 18th century by Samuel T. Sœmmerring (1798), demands it.

## Why do we consider eight pairs of cervical nerves today?

While we now consider 31 pairs of spinal nerves, this was not always the case. In the mid-16th century, Vesalius described 30 pairs of spinal nerves (Vesalius, 1555). These corresponded to seven cervical, 12 thoracic, five lumbar and six sacral vertebrae, and the suboccipital nerve passing between the occipital bone and the first cervical vertebra was joined to the last pair of cranial nerves. This description was so authoritative that it was cited in Willis (1664) and Vieussens (1684) and remained influential until the late 18th century (Monro, 1782; Winslow, 1732).

At the end of the 18th century and the beginning of the 19th, the suboccipital nerve was, in turn, considered either a cranial nerve (Bichat, 1812; Monro, 1782; Sabatier, 1791) or a spinal nerve (Asch, 1750; Bell, 1812; Haller, 1778; Portal, 1803), depending on whether the author emphasized its cranial aspect (arising from a single root of the medulla oblongata) or its spinal one (arising from the spinal cord along two roots). Since all anatomists asserted that the first thoracic nerve exits below the first thoracic vertebra because it gives rise to the first intercostal nerve, which runs under the first rib, the suboccipital nerve was considered a spinal nerve (Asch, 1750; Bell, 1812; Haller, 1778). This led to the cervical nerves, which were originally seven in number, being increased to eight, with Sœmmerring being the first to describe the eighth cervical spinal nerve.

## What was Sœmmerring's description?

Sœmmerring named the spinal nerves according to their point of issue from the vertebral column (Sœmmerring, 1798). Their segmental pattern is transferred by the somite (Keynes and Stern, 1984), which also gives rise to the vertebra, and neural arches develop in interaction with the spinal ganglia and nerves (Christ and Wilting, 1999). All originate from the spinal cord via a dorsal and a ventral root, which join and pass the vertebral column through a foramen limited by the pedicles of two adjacent vertebrae. Some of these nerves (i.e. the first seven) lie superior to the vertebrae, while others (i.e., thoracic and lumbar nerves) exit from below (Standring, 2020).

One question arises: if spinal nerves reflect the segmental organization of the nervous system and the human frame, how can somites and nerves, two structures so dependent on each other, have different relationships with the vertebral column? As the shape and composition of all spinal nerves are identical and mirror the anatomical organization of the body, the answer becomes obvious: to one somite, one nerve, one vertebra.

## Where is Sœmmerring's error?

Sœmmering seems to be wrong because he described eight cervical spinal nerves rather than seven. This discrepancy arose because the suboccipital nerve was described as a spinal nerve rather than a cranial nerve. The problem is that he did not question the origin of the first thoracic nerve, which, for ancient anatomists, would necessarily have been the origin of the first intercostal nerve.

All spinal nerves pass through an intervertebral foramen and divide into two rami in very close contact with the vertebral arch of the underlying vertebra (Lazorthes, 1971).

The first spinal nerve courses over the posterior arch of the first cervical vertebra at the level of the groove of the vertebral artery, which is homologous to the superior notch of the vertebral pedicle (Testut, 1893), especially since sometimes a thin bony spiculum placed above converts it into a complete foramen (Standring, 2020; Testut, 1893).

The second spinal nerve runs through a fairly wide space that functions as an intervertebral foramen. It consists of the very discreet inferior notch of the atlas and the pedicle of the axis, which merges with the lamina (Testut, 1893).

The next 23 spinal nerves exit the vertebral column between the superior and inferior vertebral notches and divide into two rami. The anterior ramus of the third to seventh spinal nerves lies on the *sulcus nervi spinalis* on the upper surface of the underlying transverse process, and the dorsal ramus crosses its posterior root and wraps around the cranial articular process of the same vertebra. The ramus of the eighth nerve is closely applied to the neck of the first rib (Paturet, 1964) before the posterior ramus wraps around the cranial articular process of the first thoracic vertebra (Lazorthes, 1971). In the thoracic region, the anterior rami give rise to the intercostal nerves, which run along the neck of the underlying rib. Meanwhile, the dorsal rami run posteriorly and enter a vertical osteofibrous slot limited superiorly by the overlying transverse process, inferiorly by the upper edge of the underlying rib, and medially by the underlying cranial articular process. In the same way, the dorsal rami of the lumbar nerves run posteriorly and downwards over the transverse process of the underlying vertebra and wrap around its superior articular process. The caudal spinal nerves run through the sacral canal. On each side, four T-shaped conduits separated by a sagittal bony column homologous to the pedicles allow the 26th to 29th spinal nerves to pass through the intervertebral foramen.

The last two pairs of spinal nerves exit the sacral canal through the caudal opening (Poirier and Charpy, 1899). The penultimate, more lateral pair passes in front of the horn of the sacrum, between the lateral edge of the sacrococcygeal joint and the medial bundle of the lateral sacrococcygeal ligament, which often ossifies with age. This osteofibrous passage, limited by the 5th sacral vertebra and the first coccygeal segment, serves as the penultimate intervertebral foramen (Testut, 1893). The last spinal nerve, which is very delicate, exits medially from the previous one and obliquely runs along the dorsal surface of the first coccygeal piece before passing under the dorsal ligament of the mid-coccygeal joint (Testut, 1893). The last intervertebral foramen is therefore limited by the first two pieces of the coccyx, which are converted into an osteofibrous foramen by this ligament.

Since every spinal nerve courses through a foramen and its posterior ramus wraps around the cranial articular process of the underlying vertebra, it is understood that there is a close relationship between one segment of the peripheral nervous system and the underlying segment of the vertebral column. All spinal nerves course superior to their corresponding vertebrae.

## Why does the thoracic spinal nerve run over the corresponding rib?

Like all vertebrates, the human body exhibits a segmental organization. This is mostly visible in the metameric division of the nervous system and the series of bones that form the vertebral column. The number of spinal nerves is strictly correlated to the number of vertebrae. The presence of supernumerary vertebrae leads to an increase in the number of spinal nerves, and conversely, a reduction in the number of vertebrae causes a decrease in the number of spinal nerves (Paturet, 1964).

Segmental organization is a fundamental process remarkably conserved during phylogenesis since it also exists in invertebrates (earthworms). In human embryogenesis, somites occur very early in development (Christ and Wilting, 1999). The segmental pattern is transferred by the somite to the blood vessels, spinal ganglia and nerves (Keynes and Stern, 1984) according to a hierarchy in which the somite represents the primary, the spinal ganglia and nerves the secondary and the vertebral neural arches the tertiary elements (Christ and Wilting, 1999). The metameric division of the neural tube, the vertebral column, the thorax and the ribs depends on their arrangement.

**Figure 1:**
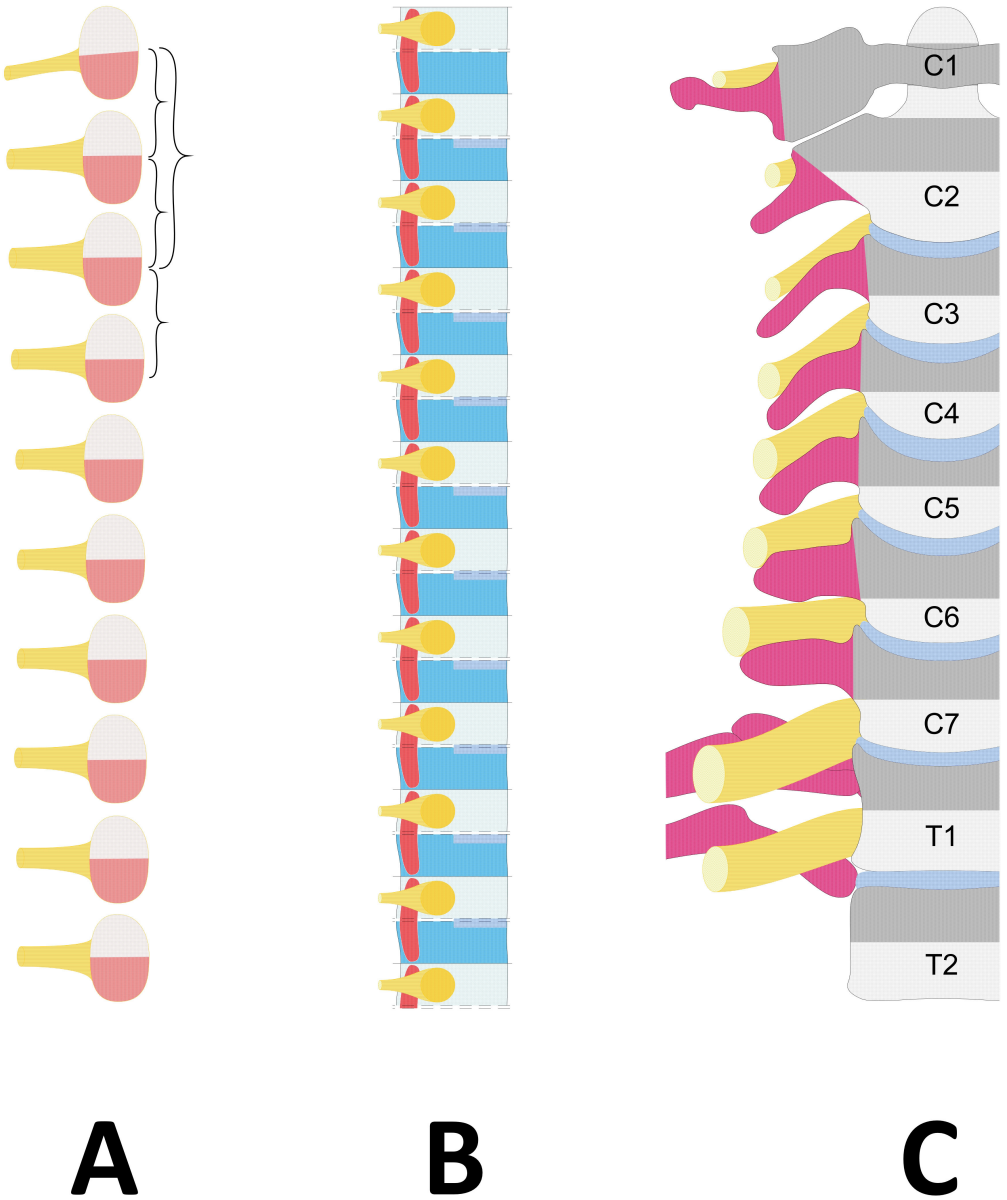
Diagram showing the embryological vertebral column development. (**A**) Stage of somites. The somite, centered by a spinal nerve (Yellow), is divided into the dorsal dermomyotome (Light pink) and the ventral sclerotome (Red). (**B**) Different development of the sclerotome with resegmentation. Axially, the fusion of the caudal part (Dark blue) with the cranial part of the following sclerotome (Light blue) forms the vertebral body lined by the disc rudiments (Middle blue). Laterally, the spinal nerve (Yellow) develops in the loose cranial half (Light blue), and the transverse process, the anterior part of the pedicle and the proximal part of the rib (Dark pink) originate from the caudal dense half (Dark blue). (**C**) Projection of the sclerotome boundaries on the differentiated vertebral column. The spinal nerve (Yellow) courses on the transverse process or proximal rib (Dark pink) of its corresponding vertebra (Gray). Dark gray corresponds to the caudal dense half of the sclerotome, and light gray corresponds to the loose cranial half of the sclerotome. Due to resegmentation, the sclerotome does not correspond to the vertebra but is centered on a vertebral disc (Middle blue).

Each pair of somites is centered by a pair of spinal nerves and connected to a segment of the spinal cord. The cells of the early somite are assigned to different compartments: the dorsal half gives rise to the dermomyotome, from which subcutaneous tissue and skeletal musculature proceed (Christ and Wilting, 1999), while the ventral half is the source of the sclerotome, which forms an important component of the axial skeleton (Figure 1A). In turn, the sclerotome can be subdivided into ventral, dorsal, lateral and central compartments, which will form the vertebral body, the main part of the neural arch, the distal part of the rib, the pedicle (including the transverse process) and the proximal part of the rib, respectively (Christ and Wilting, 1999; Scaal, 2016). Furthermore, it is divided by a transient, faint cleft that does not extend to the axial area (Ebner, 1888) into a dense caudal and a loose cranial half. The spinal nerves and ganglia develop in the loose cranial half of the sclerotome (Keynes and Stern, 1984; Remak, 1855; Scaal, 2016; Figure 1B).

While two pairs of adjacent sclerotomes contribute to the formation of the vertebral body through the fusion of their inferior part with the superior part of the next caudal sclerotome in the “so-called” phenomenon of resegmentation (Ebner, 1888), the formation of the lateral vertebral elements—especially the transverse process and the proximal part of the rib—originates from the sole dense caudal half of the sclerotome (Scaal, 2016) and takes place **without resegmentation** (Christ and Wilting, 1999; Figure 1B).

Since each developing spinal nerve forms within the loose cranial half, it lies on the dense caudal half of its sclerotome, which gives rise to the ventral part of the pedicle, the transverse process and the proximal part of the rib (the costal head and neck, including the costovertebral joint) (Scaal, 2016). In summary, a sclerotome derived from a somite is ultimately divided by an intervertebral disc and corresponds to the distal part of one vertebra (the loose cranial half of the sclerotome) and the cranial part of the following vertebra, including its transverse process or proximal rib (the dense caudal part of the sclerotome). Therefore, regardless of the level of the vertebral column, the spinal nerve corresponding to a vertebra is the one associated with the transverse process or the neck of the rib on which it courses (Figure 1C).

## Why cannot there be eight pairs of cervical nerves?

The location of the boundary between the head and neck seems to vary according to different authors (Christ and Wilting, 1999; Padget, 1954; Müller and O'Rahilly, 2003; O'Rahilly and Müller, 2003; Scaal, 2016; Wilting et al., 1995). Nevertheless, the occipital bone should be considered homologous to the vertebrae and included as the most cranial part of the vertebral column because it derives from the fusing sclerotomes of the first four or five somites (Christ and Wilting, 1999; Müller and O'Rahilly, 2003; O'Rahilly and Müller, 2003; Scaal, 2016).

The Atlas, as the first vertebral segment, lies within the first spinal somite (Christ and Wilting, 1999; Padget, 1954; Müller and O'Rahilly, 2003; O'Rahilly and Müller, 2003; Scaal, 2016). Its cranial half gives rise to the proatlas, which will later be incorporated into the apical part of the dens axis, when the caudal half fuses with the cranial part of the underlying somite to form the dens axis (the vertebral body of atlas), which later fuses with the vertebral body of the second vertebra, the axis (Christ and Wilting, 1999). Since the first spinal nerve centers the first spinal somite, it is located in the dense zone of the first sclerotome. The second spinal nerve runs along the dense caudal zone of the second spinal sclerotome at the origin of the neural arch of the axis. Once it is understood that each spinal nerve runs over its corresponding vertebra—for example, the eighth spinal nerve with the eighth vertebra, which is nothing else than the first thoracic vertebra—the current naming convention, such as ‘eighth cervical spinal nerve', becomes inadmissible.

## Why are there two coccygeal nerves?

Since there are 31 pairs of spinal nerves, if the eighth pair of spinal nerves is the first thoracic nerve, there must be two pairs of coccygeal nerves.

The neural tube extends longitudinally along the axis of the embryo. Although there can be up to 38 or 39 pairs of somites (O'Rahilly et al., 1990), several disappear in relation to the caudal regression of the human embryo. In some instances, rudiments of coccygeal nerves still remain detectable (O'Rahilly and Müller, 2003). The most caudal part of the primitive spinal cord, along with the pia mater, arachnoid mater and dura mater, corresponds to the very end of the vertebral column and, in most cases, attaches to the fifth coccygeal vertebra (O'Rahilly et al., 1990). The spinal nerves emerge perpendicularly through their own intervertebral foramina, and there is no *cauda equina*. As the vertebral column elongates, the spinal cord ascends, and the caudal spinal nerves become stretched vertically until they reach their foramina to exit. The *cauda equina* then appears. Arising at the tip of the *conus medullaris*, the *filum terminale* attaches to the posterior surface of the first two coccygeal segments (Christ and Wilting, 1999). This caudal insertion marks the position of the lower end of the definitive spinal cord, and the last spinal nerve (the 31st) faces the second coccygeal vertebra. Effectively, in the adult, the last two pairs of spinal nerves exit the vertebral column through the sacral hiatus. The penultimate, more lateral pair crosses the sacral horns and passes through an osteofibrous slot. The latter serves as a vertebral pedicle, being limited by the fifth sacral vertebra and the first coccygeal piece, especially when a ligament that often ossifies with age converts it into a complete penultimate foramen. The last pair runs along the dorsal surface of the first coccygeal piece before exiting laterally under the dorsal ligament of the mid-coccygeal joint (Keynes and Stern, 1984). This last intervertebral foramen, converted into an osteofibrous foramen by this ligament, is limited superiorly by the first piece of the coccyx and inferiorly by the second. Obviously, the last two spinal nerves exit the vertebral column, each above a different coccygeal piece. The first coccygeal nerve courses on the first coccygeal vertebra, and the second nerve courses on the second vertebra through a foramen.

## Conclusion

According to these anatomical and embryological arguments, the thoracic nerve runs over its corresponding rib, the eighth spinal nerve corresponds to the first thoracic nerve, and there are two coccygeal nerves. The eighth cervical nerve does not exist. This implies that the metamere of the spinal cord and the 31 pairs of spinal nerves, which innervate striated muscles (myotomes) and skin (dermatomes), must be arranged as follows: seven cervical, 12 thoracic, five lumbar, five sacral and two coccygeal.

## References

[B1] Asch G. T. (1750). Dissertatio Inauguralis de Primo Pare Nervorum Medullæ Spinalis. Officina Vendenhoekiana.

[B2] Bell J. (1812). The Anatomy of the Human Body, vol III. New York, NY: Collins and Co.

[B3] Bichat X. (1812). Anatomie Descriptive. Nouvelle ed. Paris: J.S. Chaudé, Gabon.

[B4] Christ B. Wilting J. (1999). From somites to vertebral column. Ann. Anat. 174, 23–32. 10.1016/S0940-9602(11)80337-7 1605355

[B5] Ebner V. V. (1888). Urwirbel und Neugliederung der Wirbelsäule. Sitzungsber. Akad.Wiss III*/97*, 194–206.

[B6] Haller A. (1778). De Partium Corporis Humani. Præcipuarum Fabrica et Functionibus. Opus Quinquaginta Annorum tom VIII Cerebrum Nervi. Ex Prelis Sociatatum Typographicarum.

[B7] Keynes R. J. Stern C. D. (1984). Segmentation in the vertebrate nervous system. Nature 310, 786–789. 10.1038/310786a0 6472458

[B8] Lazorthes G. (1971). Le Système Nerveux Périphérique. Description. Systématisation. Exploration. Masson et Cie.

[B9] Monro A. (1782). The Anatomy of the Human Bones, Nerves and Lacteal Sac and Duct, eds. *W. Gordon, J. Dickson*. Edinburgh. 10.5962/bhl.title.114861

[B10] Müller F. O'Rahilly R. (2003). Segmentation in staged human embryos: the occipitocervical region revisited. J. Anat. 203, 297–315. 10.1046/j.1469-7580.2003.00219.x 14529047 PMC1571167

[B11] O'Rahilly R. Müller F. (2003). Somites, Spinal Ganglia, and Centra. Enumeration and Interrelationships in Staged Human Embryos, and Implications for Neural Tube Defects. Cells Tissues Organs 173, 75–92. 12649586 10.1159/000068948

[B12] O'Rahilly R. Müller F. Meyer D. B. (1990). The human vertebral column at the end of the embryonic period proper. 4. The sacrococcygeal region. J. Anat. 168, 95–111. 2182589 PMC1256893

[B13] Padget D. H. (1954). Designation of the embryonic intersegmental arteries in reference to the vertebral artery and subclavian stem. Anat. Rec. 119, 349–356. 10.1002/ar.1091190306 13197795

[B14] Paturet G. (1964). Traité D'anatomie Humaine, Tome IV Système Nerveux. Masson et Cie.

[B15] Poirier P. Charpy A. (1899). Traité D'anatomie Humaine, Tome 3 Système Nerveux: les nerfs. Masson et Cie.

[B16] Portal A. (1803). Cours D'anatomie Médicale Ou Élémens de L'anatomie De L'homme, Tome Quatrième. Paris: Baudouin.

[B17] Remak R. (1855). Untersuchungen über die Entwicklung der Wirbeltiere. G Reimer.

[B18] Sabatier R. (1791). Traité Complet D'anatomie ou Description de Toutes le Parties du Corps Humain, Tome III. 3ème ed. Paris: Théophile Barrois le Jeune.

[B19] Scaal M. (2016). Early development of the vertebral column. Semin. Cell. Dev. Biol. 49, 83–91. 10.1016/j.semcdb.2015.11.003 26564689

[B20] Sœmmerring S. T. (1798). De Corporis Humani Fabrica, Tomus Quartus: De Cerebro et de nervis. *Varrentrappii et Wenneri*.

[B21] Standring S. (2020). Gray's Anatomy: The Anatomical Basis of Clinical Practice. Philadelphia: Elsevier.

[B22] Testut L. (1893). Traité D'anatomie Humaine: Anatomie Descriptive - Histologie - Développement, 3 Tomes. Octave Douin.

[B23] Vesalius A. (1555). De Humani Corporis Fabrica Libri Quartus. Basileae: Johannes Oporinus.

[B24] Vieussens R. (1684). Nevrographia Universalis. Lugduni: Joannem Certe.

[B25] Willis T. (1664). Cerebri Anatome: Cui Accessit Nervorum Descriptio Et Usus, Studio Thomæ Willis. Typis Ja Flesher, impensis Jo. Martyn and Ja. Allestry.

[B26] Wilting J. Ebensperger C. Müller T. S. Koseki H. Wallin J. Christ B. (1995). Pax-1 in the development of the cervicooccipital transitional zone. Anat. Embryol. 192, 221–227. 10.1007/BF00184746 8651506

[B27] Winslow J. B. (1732). Exposition Anatomique De La Structure Du Corps Humain. Guillaume Desprez, Jean Desessartz.

